# Hybrid Fine-Tuning Strategy for Few-Shot Classification

**DOI:** 10.1155/2022/9620755

**Published:** 2022-10-08

**Authors:** Lei Zhao, Zhonghua Ou, Lixun Zhang, Shuxiao Li

**Affiliations:** ^1^Institute of Automation, Chinese Academy of Sciences, Beijing, China; ^2^University of Electronic Science and Technology of China, Chengdu, China

## Abstract

Few-shot classification aims to enable the network to acquire the ability of feature extraction and label prediction for the target categories given a few numbers of labeled samples. Current few-shot classification methods focus on the pretraining stage while fine-tuning by experience or not at all. No fine-tuning or insufficient fine-tuning may get low accuracy for the given tasks, while excessive fine-tuning will lead to poor generalization for unseen samples. To solve the above problems, this study proposes a hybrid fine-tuning strategy (HFT), including a few-shot linear discriminant analysis module (FSLDA) and an adaptive fine-tuning module (AFT). FSLDA constructs the optimal linear classification function under the few-shot conditions to initialize the last fully connected layer parameters, which fully excavates the professional knowledge of the given tasks and guarantees the lower bound of the model accuracy. AFT adopts an adaptive fine-tuning termination rule to obtain the optimal training epochs to prevent the model from overfitting. AFT is also built on FSLDA and outputs the final optimum hybrid fine-tuning strategy for a given sample size and layer frozen policy. We conducted extensive experiments on mini-ImageNet and tiered-ImageNet to prove the effectiveness of our proposed method. It achieves consistent performance improvements compared to existing fine-tuning methods under different sample sizes, layer frozen policies, and few-shot classification frameworks.

## 1. Introduction

Deep learning has recently attracted attention due to its outstanding performances in computer vision (e.g., image classification and object detection), NLP, and reinforcement learning. In the military domain, unmanned aerial vehicles (UAVs) play a significate role in jamming and reconnaissance. Bai et al. [1] established a 3D UAV air combat model and a UAV maneuvering decision algorithm based on deep reinforcement learning to achieve autonomous operation of UAVs in the future. Saqlain et al. [2] applied deep learning and computer vision to retail management to boost retail sales, proposing a hybrid approach that can effectively monitor retail shelves and satisfy planograms. In face recognition systems, Yang and Song [3] improved the face recognition effect in different light intensities combined with the deep learning algorithm, which is of great practical value.

The success of deep learning is mainly attributed to the following three factors, i.e., powerful computing resources, complex network frameworks, and large-scale datasets. However, obtaining sufficient labeled data in many application scenarios, such as rare diseases, new species, and defective industrial products, is difficult or even impossible. When the annotated data are scarce, traditional deep learning methods generally perform unsatisfactorily. Considering that humans can rapidly establish cognition to novel concepts from just a single or a handful of examples, we hope the network can acquire the ability to recognize visual objects for novel classes with high accuracy and generalization by learning from only a few samples.

Towards the goal of shrinking the gap between human intelligence and artificial intelligence, few-shot learning, especially few-shot classification (FSC), was proposed. FSC aims to learn an effective classifier from the target dataset, which only contains a few labeled images for novel classes. However, different from general deep learning, it is impossible to train an effective classification model from scratch only using the target dataset due to its limited capacity. Therefore, current FSC methods usually employ a base dataset, which contains abundant labeled images for base classes and has no category intersection with the target dataset. The model is firstly pretrained on the base dataset to learn a feature extractor and then is transferred to the target domain for fine-tuning to boost the performance of FSC. At the pretraining stage, the feature extractor is pretrained either on the base dataset directly or by meta-learning which constructs massive few-shot tasks to imitate the target scenarios. As for the fine-tuning stage, current methods always choose the fine-tuning settings relying on experience, e.g., how to set the learning rate, which layers are selected to be frozen, and how many training epochs to be set. They prefer to set the learning rate as 0.001 [4, 5], usually select linear probing (updating only the last linear layer) [6] or full fine-tuning (updating all the model parameters) [7–9], and rarely mention how many training epochs are set. Since there are no validation and test images in the target dataset, it is impossible to evaluate the performance of the fine-tuned model, so how to set hyperparameters beyond experience remains a problem. In addition, the classifier parameters will also be quickly converged to a nonoptimal solution under few-shot conditions, which further reduces the classification performance.

**Figure 1:**
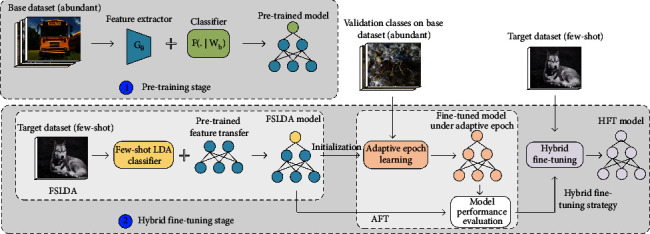
Main idea and flowchart of the proposed HFT method for FSC. HFT performs the fine-tuning process based on the pretrained model. It includes an FSLDA module and an AFT module. FSLDA constructs the optimal linear classifier under the few-shot conditions to get the FSLDA model. AFT executes adaptive epoch learning and model performance evaluation using the validation classes of the base dataset to obtain the hybrid fine-tuning strategy, which is finally adopted for fine-tuning the pretrained model using the target dataset to get the HFT model.

To address the problems mentioned above, in this work, we propose a hybrid fine-tuning strategy (HFT) for FSC, as shown in Figure 1. We first pretrain on the base dataset to get the pretrained model and then fine-tune it on the target dataset according to the acquired hybrid fine-tuning strategy by HFT. The proposed HFT includes an FSLDA module and an AFT module. FSLDA constructs the optimal linear classifier by fully excavating the professional knowledge of the target dataset, which provides the last fully connected layer of the pretrained model a better starting point that fine-tuning with backpropagation probably cannot reach, thus guaranteeing the lower bound of the model accuracy. AFT executes adaptive epoch learning using the validation classes of the base dataset by designing an adaptive fine-tuning termination rule to obtain the optimal training epochs. Therefore, AFT sets hyperparameters by learning instead of experience and can prevent the model from overfitting. AFT also implements model performance evaluation to obtain the hybrid fine-tuning strategy. Finally, we update the pretrained model with the acquired hybrid fine-tuning strategy using the target dataset to get the HFT model. In summary, the main contributions of this study are as follows:

1. We improve linear discriminant analysis for FSC and propose the FSLDA module, which can be used to initialize the last fully connected layer parameters of the pretrained model and guarantees the lower bound of the model accuracy. Ablation studies on mini-ImageNet dataset show that the Meta-Baseline method [10] with the FSLDA module alone has an average performance improvement of 3.07% and 2.99% under the layer frozen policy “Last1” and “All,” respectively.
2. We introduce adaptive epoch learning to the fine-tuning stage and propose the AFT module, which can prevent the model from overfitting and output the hybrid fine-tuning strategy under different sample sizes and different layer frozen policies. Ablation results on mini-ImageNet dataset show that the Meta-Baseline method [10] with AFT under the layer frozen policy “All” further brings 0.40%, 0.99%, and 0.79% performance improvements for sample sizes of 10-shot, 20-shot, and 30-shot, respectively.
3. The acquired hybrid fine-tuning strategy is evaluated under three recently proposed few-shot classification methods. Comparative experiments show that the proposed HFT has an average performance improvement of 2.30% on the mini-ImageNet dataset and 2.78% on the tiered-ImageNet dataset over current experience-based finetuning methods.

## 2. Related Works

### 2.1. Few-Shot Classification

Currently, many works have been proposed to address FSC [11–19], which can be mainly divided into three categories: initialization-based methods, metric-based methods, and hallucination-based methods. Initialization-based methods use the target dataset to fine-tune the pretrained model with a small number of gradient backpropagation steps [20, 21]. Metric-based methods extract features from both the labeled and unlabeled images and predict the class labels by computing the similarity metric function, such as cosine similarity [22], Euclidean distance [23], and relation modules [24]. Hallucination-based methods [25] focus on data augmentation by learning a generator from the base dataset and applying it to novel classes to expand the capacity of the target dataset. Recently, some works have employed self-supervision [26, 27], knowledge distillation [28, 29], and distribution calibration [30, 31] to strengthen the feature extractor or the last classifier. Our work is built on the metric-based pretraining methods and improves the initialization-based fine-tuning methods by introducing a hybrid fine-tuning strategy.

### 2.2. Fine-Tuning Strategy

Before fine-tuning the model with the target dataset, key hyperparameters need to be set, such as the layer frozen policy, the learning rate, and the training epochs. Due to the scarcity of the target dataset, we cannot judge whether the model is suboptimal, overfitted, or underfitted. Thus, current methods usually set the above hyperparameters by experience. There are two popular strategies for the layer frozen policy: running gradient descent on all model parameters [7–9] and fine-tuning the head but freezing lower layers [32]. Some works [33, 34] claim that fine-tuning all model parameters leads to better accuracy than only fine-tuning the head, while most researchers have no consistent conclusions about this. For the learning rate, the mainstream methods [35, 36] on FSC select to set it as 0.001. As for the training epochs, current methods use fixed settings, and their value is rarely mentioned. Recently, an evolutionary algorithm [37] has been proposed for searching the best finetuning configuration, focusing on the learning rate and the layer frozen policy. Our work emphasizes learning the best training epochs, which is essential to prevent the model from overfitting or underfitting and is complementary to the work in [37]. In addition, we propose the FSLDA module to construct the optimal linear classifier for FSC to avoid suboptimal solutions.

## 3. Methods

This section first introduces the preliminary foundations, including problem definition and model pretraining for FSC. We then give the technical details for the FSLDA and AFT modules, respectively.

### 3.1. Preliminary Foundations

#### 3.1.1. Problem Definition

In the standard FSC task, we generally have a base dataset *𝒟*_*b*_ and a target dataset *𝒟*_*n*_. Generally, *𝒟*_*b*_ contains abundant labeled samples for base classes, while *𝒟*_*n*_ has only a few labeled samples for novel classes (usually 1 to 30 for each class). Denote *𝒞*_*b*_ and *𝒞*_*n*_ as the category spaces of base classes and novel classes, respectively, which are nonoverlapping, i.e., {*𝒞*_*b*_∩*𝒞*_*n*_}=∅. Let *𝒩*_*b*_ and *𝒩*_*n*_ denote the number of samples in the base and the target datasets, respectively. With these definitions, *𝒟*_*b*_ and *𝒟*_*n*_ can be further denoted as *𝒟*_*b*_={(*x*_*i*_, *l*_*i*_)*|l*_*i*_ ∈ *𝒞*_*b*_}_*i*=1_^*𝒩*_*b*_^ and *𝒟*_*n*_={(*x*_*j*_, *l*_*j*_)*|l*_*j*_ ∈ *𝒞*_*n*_}_*j*=1_^*𝒩*_*n*_^, where *x* represents the sample in the dataset and *l* indicates the label that the sample was annotated with. The goal of FSC is to train models with *𝒟*_*b*_ and *𝒟*_*n*_ for predicting the labels of samples in the test dataset of novel classes. Specifically, considering a *C*-way *K*-shot metric-based meta-learning FSC task, massive meta-learning tasks, each of which includes a support set and a query set, are randomly sampled from the base dataset to imitate the target task. The support set consists of *C* classes with *K* labeled samples in each class, and the corresponding query set has the same classes as the support set, each of which has *Q* unlabeled samples. The goal of metric-based meta-learning is to update the model to predict the labels of the *C* × *Q* samples in the query set by computing their similarities to the support set. Through continuous learning from massive meta-learning tasks, the pretrained model can memorize more scene knowledge and thus has better generalization ability for FSC tasks.

#### 3.1.2. Model Pretraining

A fundamental step for FSC is pretraining the model on the base dataset to provide a suitable feature extractor *G*_*θ*_. Specifically, the model is firstly trained with standard cross-entropy loss on the base dataset for all the classes to get the initialized model. Then, metric-based meta-learning is performed to continually train the model by building massive *C*-way *K*-shot tasks, finally outputting the pretrained model. This scheme can help the model improve its stability and generalization ability by imitating the few-shot settings that will be encountered in the target task. In fact, the proposed fine-tuning method in this study only uses the parameters of the pretrained model, which has nothing to do with the pretraining method. Thus, other pretraining methods based on different theories are also applicable.

### 3.2. Few-Shot LDA Module

**Figure 2:**
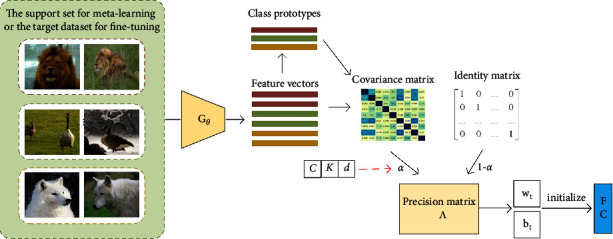
Diagram of the proposed FSLDA module. Given a *C*-way *K*-shot support set or target dataset, we first get the feature vector for each sample, the prototype for each class, and the covariance matrix for all feature vectors sequentially. Then, the rank factor *α* is introduced to obtain the precision matrix Λ for FSC tasks based on the weighted mean of the covariance matrix and the identity matrix. Finally, we obtain the parameter value of the last fully connected layer by Λ and initialize it.

Linear discriminant analysis (LDA) is a dimensionality reduction technique for supervised learning and is mainly used for classification. The core idea of LDA is to project high-dimensional data samples into the best vector space so that interclass distances are larger and intraclass distances are smaller in the new subspace. LDA needs to calculate the covariance matrix using the feature vectors of data samples in the support set or the target dataset. For FSC tasks, the feature dimension is usually larger than the number of data samples; thus, the covariance matrix is irreversible. To address this issue, FSLDA is proposed to initialize the head of the pretrained model by constructing the optimal linear classification function under few-shot conditions. As shown in Figure 2, we introduce the rank factor *α*, which is related to the feature dimension *d* and the number of data samples, to illustrate the reliability of the covariance matrix. Based on the rank factor *α*, the weighted mean of the covariance matrix and the identity matrix is computed instead to obtain the precision matrix so that the invertible condition can be satisfied. By doing so, we get the optimal solution of the FSLDA classifier, which fully excavates the professional knowledge of the given tasks.

Formally, the CNN model we train can be expressed as *y*_*i*_=*F*(*G*(*x*_*i*_)), where *x*_*i*_ is the input sample and *y*_*i*_ is the predicted class label. We decompose the network into two nested functions: the feature extractor denoted as *G*(·*|θ*_*G*_) and the last fully connected layer denoted as *F*(·*|θ*_*F*_). The goal of FSLDA is to initialize the parameters *θ*_*F*_ of *F*(·*|θ*_*F*_), which can be formulated as

(1) $$\begin{array}{c} F\left(G\left(x_{i}\right)\right)=Wv+b\text{,} \end{array}$$

where *v* ∈ *ℝ*^*d*^ denotes the output of feature extractor *G*(·*|θ*_*G*_) for the input sample *x*_*i*_, *W* ∈ *ℝ*^*c*×*d*^ and *b* ∈ *ℝ*^*c*^ are, respectively, the weight matrix and the bias vector of *F*(·*|θ*_*F*_), *d* is the output dimension of feature extractor *G*(·*|θ*_*G*_), and *c* is the number of classes.

According to the LDA theory (details are shown in the Appendix section), given a *C*-way *K*-shot task, the optimal linear classifier for class *t* is given by

(2) $$\begin{array}{c} f_{t}\left(v\right)=\mu_{t}^{T}\Sigma^{-1}v-\frac{1}{2}\mu_{t}^{T}\Sigma^{-1}\mu_{t}\text{,}\\ \mu_{t}=\frac{1}{K}\overset{K}{\underset{i=1}{\sum }}G\left(x_{t}^{i}\right)\text{,}\\ \Sigma =\frac{1}{C\cdot \left(K-1\right)}\overset{K}{\underset{i=1}{\sum }}\overset{C}{\underset{t=1}{\sum }}\left[G\left(x_{t}^{i}\right)-\mu_{t}\right]\cdot {\left[G\left(x_{t}^{i}\right)-\mu_{t}\right]}^{T}\text{,} \end{array}$$

where *x*_*t*_^*i*^ denotes the *i*th sample for the *t*th class, *μ*_*t*_ is the mean feature vector (also called the prototype) for class *t*, and Σ is the covariance matrix of the whole dataset. It can be seen that the rank of the covariance matrix Σ is *C* · (*K* − 1) for nonlinear data samples, which is usually smaller than the feature dimension *d*. Thus, the covariance matrix is irreversible and LDA cannot be directly used for FSC tasks.

To this end, we compute the precision matrix Λ directly based on the covariance matrix Σ by harmonic weighting, i.e.,

(3) $$\begin{array}{c} \Lambda ={\left[\alpha \cdot \Sigma +\left(1-\alpha \right)\cdot I\right]}^{-1}\text{,}\\ \alpha =1-ReLU\left(1-\frac{C\cdot \left(K-1\right)}{d}\right)\text{,} \end{array}$$

where *I* ∈ *ℝ*^*d*×*d*^ is the identity matrix and *α* is the rank factor to measure the reliability of the covariance matrix Σ, making the precision matrix Λ both reversible and informative. When *K* equals 1, *α* gets the value of 0 and FSLDA degenerates into prototype initialization. For non-FSC tasks (*K* is sufficiently large), *α* gets the value of 1 and FSLDA degenerates into LDA. Thus, prototype initialization and LDA are special cases of FSLDA.

Once the precision matrix Λ is available, FSLDA classifier can be constructed as

(4) $$\begin{array}{c} f_{t}\left(v\right)=\mu_{t}^{T}\Lambda v-\frac{1}{2}\mu_{t}^{T}\Lambda \mu_{t}\text{.} \end{array}$$

Finally, we use FSLDA classifier to compute *w*_*t*_, i.e., the rows of *W*, and *b*_*t*_, i.e., the individual elements of *b*, as

(5) $$\begin{array}{c} w_{t}=\mu_{t}^{T}\Lambda \text{,}\\ b_{t}=-\frac{1}{2}\mu_{t}^{T}\Lambda \mu_{t}\text{.} \end{array}$$

The FSLDA enables to initialize the parameters in *F*(·*|θ*_*F*_) by computing the precision matrix Λ of the samples in novel classes before fine-tuning, which gives the model a better initial point than random initialization. By leveraging the knowledge of samples in novel classes and optimizing it for the classifier, the FSLDA ensures a lower bound on the model's performance and makes the model converge quickly for the fine-tuning stage.

### 3.3. Adaptive Fine-Tuning Module

Drawing on the experience of meta-learning-based pretraining methods, we propose the AFT module to obtain the hybrid finetuning strategy. AFT firstly performs adaptive epoch learning using the idea of “chunk by chunk” on the validation classes of the base dataset, which evaluates the model's performance for each chunk and establishes an adaptive termination rule to output an adaptive epoch that needs to be set at the fine-tuning stage. Then, the higher one between the FSLDA model and the adaptive fine-tuned model is retained, and the optimal hybrid epoch is acquired. Finally, the above procedures are executed on massive pseudofine-tuning tasks to output the final hybrid fine-tuning strategy, ensuring that most tasks converge to higher performance.

**Figure 3:**
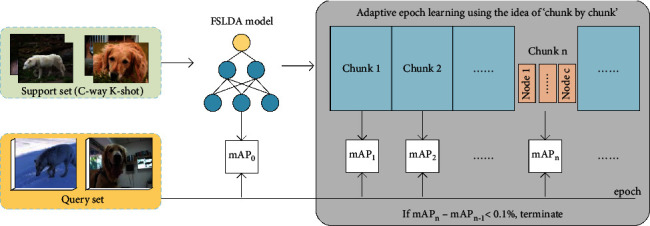
Illustration of adaptive epoch learning. Once the FSLDA model is available, we fine-tune it with the support set using the idea of “chunk by chunk” and get the corresponding sequential mAP with the query set. The fine-tuning process terminates if the accuracy gain is negligible. Note that adaptive epoch learning runs on the validation classes of the base dataset.

Specifically, massive pseudofine-tuning tasks, each of which includes a support set and a query set, are randomly sampled from the validation classes of the base dataset to imitate the fine-tuning task. Like metric-based meta-learning, the support set here is also of the *C*-way *K*-shot style. All the remaining samples in the selected classes are used as the query set to evaluate the performance of the model. As shown in Figure 3, we first use the support set to get the FSLDA model and obtain its accuracy mAP_*m*_^0^ using the query set. During adaptive epoch learning, we divide the maximum allowable epochs into *N* chunks, and each chunk contains *c* nodes. To improve the learning speed, only the model at the last epoch in each node is evaluated by the query set to get its accuracy. We regard the mean of all nodes' performance in a chunk as a representation of the chunk's performance, so as to get the macrochange trend of the accuracy curve. For the *m*th pseudofine-tuning task, we can get its “chunk by chunk” performance series, denoted as {mAP_*m*_^0^,…, mAP_*m*_^*b*^, mAP_*m*_^*b*+1^, mAP_*m*_^*n*^, ⋯}, where *b* is the starting evaluation chunk index to avoid disturbances at the initial fine-tuning stage. The process terminates if the accuracy gain is negligible and outputs the adaptive chunk index:

(6) $$\begin{array}{c} Iter_{m}=\underset{n}{min}\left\{mAP_{m}^{n}-mAP_{m}^{n-1}<0.1\%\right\},n\in \left[b,N\right]\text{.} \end{array}$$

Then, we combine the advantages of the FSLDA model and the adaptive epoch learning and set the optimal hybrid epoch as

(7) $$\begin{array}{c} epoch_{m}=\left\{\begin{array}{cc} a\cdot Iter_{m}\text{,} & mAP_{m}^{Iter_{m}}>mAP_{m}^{0}\text{,}\\ 0\text{,} & otherewise\text{,} \end{array}\right\}, \end{array}$$

where *a* is the number of epochs contained in a chunk.

When the optimal hybrid epochs for *M* pseudofine-tuning tasks are ready, the optimal hybrid finetuning strategy can be finally acquired by

(8) $$\begin{array}{c} epoch=\left\{\begin{array}{cc} Quantile\left(\left\{epoch_{m}\right\},0.9\right)\text{,} & M^{\prime }>\frac{M}{2}\text{,}\\ 0\text{,} & otherwise\text{,} \end{array}\right. \end{array}$$

where *M*′=∑_*m*=1_^*M*^1(epoch_*m*_) indicates the number of tasks needing to be fine-tuned, and 1 is the indicator function. When most pseudofine-tuning tasks do not need the fine-tuning stage (epoch=0), the optimal hybrid fine-tuning strategy adopts FSLDA as the final strategy. Otherwise, it uses the 0.9 quantile of the optimal hybrid epochs to ensure that most tasks can be converged. In the latter case, the optimal hybrid fine-tuning strategy performs both FSLDA and AFT.

The pipeline for AFT is summarized as Algorithm 1.

## 4. Experiments

In this section, we first briefly describe the experimental setup. Then, HFT experiments are carried out to give the hands-on hybrid fine-tuning strategy under different sample sizes and layer frozen policies. Finally, extensive comparison and ablation experiments on the benchmark datasets are conducted to demonstrate the effectiveness of our strategy.

### 4.1. Experimental Setup

#### 4.1.1. Dataset

We employ mini-ImageNet [22] and tiered-ImageNet [38] datasets. Mini-ImageNet is a subset of ImageNet. It consists of 100 classes, and each class has 600 images with a size of 84 × 84. We follow the setting proposed by [39] to split the datasets into 64, 16, and 20 classes as the training, validation, and testing sets, respectively. Tiered-ImageNet is a larger subset of ImageNet than mini-ImageNet. It has 608 classes, and each class contains 1,281 images on average. In the experiment, 351, 97, and 160 classes are selected as the training, validation, and test set stemming from 20, 6, and 8 superclasses, respectively.

#### 4.1.2. Implementation Details

Following the settings in [10], for the pretraining stage, we first train 100 epochs with batch size 128 on mini-ImageNet, and the learning rate decays at epoch 90. We use SGD optimizer with momentum 0.9, the learning rate 0.1, the decay factor 0.1, and the weight decay 0.0005. For the meta-learning stage, we use SGD optimizer with the weight decay 0.0005 and the learning rate 0.001. For the fine-tuning stage, we set up two kinds of layer frozen policies following [40], namely, fine-tuning all layers (“All,” updating all parameters of the model) and fine-tuning the last layer (“Last1,” allowing to update only the last fully connected layer of the model). We use the SGD optimizer with momentum 0.9, the weight decay 0.0005, and the learning rate 0.001. We use ResNet-18 as the backbone network and apply standard data augmentation, including random resized crop and random horizontal flip.

**Figure 4:**
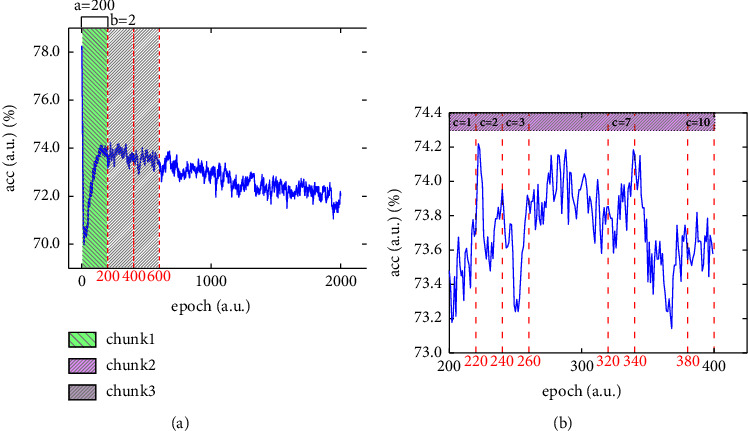
Typical accuracy curve to illustrate the hyperparameter settings for AFT.

For the hyperparameter *M*, we refer to related work [37] and follow the general meta-learning configurations, setting the total number of pseudofine-tuning tasks *M*=100. As for the maximum number of epochs *E*^max^, we find that the maximum value of the optimal epoch does not exceed 2000. Therefore, we set *E*^max^=2000 to save computing resources. As per Figure 4(a), the accuracy curve has short-term vibration at the beginning and returns to normal before the epoch around 200. So, we set the number of epochs contained in a chunk *a*=200 and the starting chunk number *b*=2 to make the adaptive algorithm avoid the influence of short-term vibration during the initial fine-tuning stage. According to Figure 4(b), we see a slight variation in accuracy within a chunk. In order to get the balance between estimation accuracy and calculation efficiency, we set the number of nodes contained in a chunk *c*=10, only evaluating the model 10 times for each chunk.

### 4.2. HFT Experiments

Following Algorithm 1, we perform experiments on mini-ImageNet to give the hands-on hybrid fine-tuning strategy under different sample sizes (1, 5, 10, 20, 30) and different layer frozen policies (“Last1,” “All”).

The main results are shown in Table 1. For the layer frozen policy “Last1,” the optimal adaptive epoch is always 0 under different sample sizes, which means the FSLDA has initialized the head of the pretrained model so well that only fine-tuning the last layer cannot make the model achieve better performance. Thus, the hands-on hybrid fine-tuning strategy under the layer frozen policy “Last1” is only FSLDA that has constructed the optimal solution for the classifier. In this case, further fine-tuning may lead to suboptimal solutions. In contrast, the hands-on hybrid fine-tuning strategy is inconsistent for the layer frozen policy “All” under different sample sizes. For sample sizes of 1-shot and 5-shot, the hands-on hybrid fine-tuning strategy is also only FSLDA. A common assumption is that too few samples in the support set are not enough to update all the model parameters for better performance. While for sample sizes of 10-shot, 20-shot, and 30-shot, the optimal adaptive epoch is no longer 0. Moreover, as the sample size increases, the optimal adaptive epoch increases, but it is always smaller than the maximum number of epochs. Thus, the hands-on hybrid fine-tuning strategy for sample sizes of 10-shot, 20-shot, and 30-shot contains both FSLDA and AFT. This indicates that adaptive fine-tuning can achieve better performance under the layer frozen policy “All” as the sample size increases.

**Figure 5:**
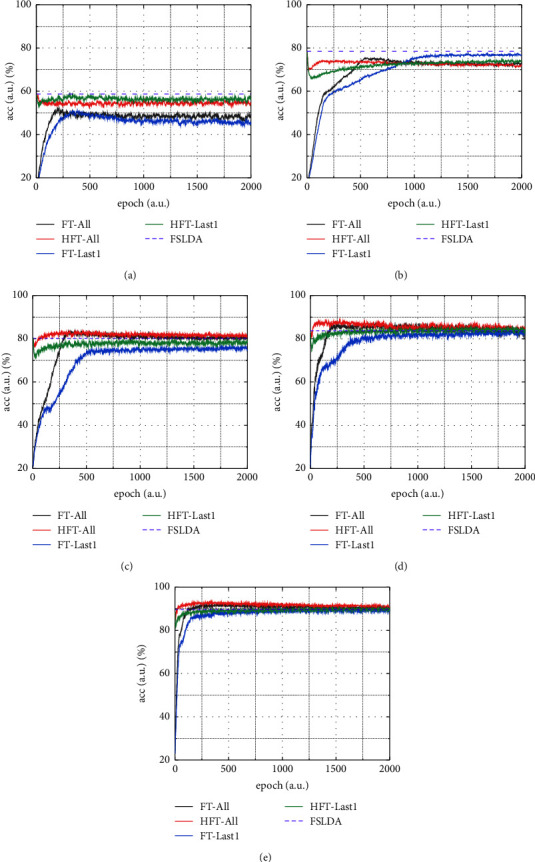
Typical convergence curves of testing accuracy during adaptive epoch learning on mini-ImageNet for sample sizes of 1-shot (a), 5-shot (b), 10-shot (c), 20-shot (d), and 30-shot (e).

Furthermore, Figure 5 shows typical convergence curves of testing accuracy during adaptive epoch learning on mini-ImageNet under different layer frozen policies and sample sizes. Here, FT-All and FT-Last1, respectively, refer to updating all parameters of the model and updating only the head, where the head is initialized randomly and the fixed epoch is set by experience. HFT-All and HFT-Last1 refer to performing fine-tuning under the corresponding layer frozen policies “All” and “Last1,” where the head is initialized by FSLDA and the epoch is set according to the acquired hands-on hybrid fine-tuning strategy. FSLDA refers to testing accuracy of the FSLDA model without fine-tuning. Note that we show the full curves for HFT-All and HFT-Last1 in Figure 5 for better comparison. We can see that, for sample sizes of 1-shot and 5-shot, the performance of the FSLDA model (purple dotted horizontal line) is always better than those of other methods, indicating that FSLDA is enough when the sample size is no more than 5. While for sample sizes of 10-shot, 20-shot, and 30-shot, the FSLDA model outperforms FT-Last1 (blue lines) and HFT-Last1 (green lines) but is not as good as FT-All (black lines) and HFT-All (red lines) and the latter one is slightly better. These all indicate the reasonableness of the acquired hands-on hybrid fine-tuning strategy.

### 4.3. Comparative Experiments

Based on the hands-on hybrid fine-tuning strategy obtained in Section 4.2, we now compare the performance of the hybrid fine-tuning strategy (HFT-Last1/HFT-All) with that of the traditional fine-tuning strategy (FT-Last1/FT-All) under different pretraining methods including RFS-simple [29], SKD-GEN0 [41], and R2D2 [42]. For the sake of fairness, the training epoch for FT-Last1/FT-All is set as *E*^max^, i.e., the hyperparameter in Algorithm 1, and other parameter settings are consistent with those of HFT-Last1/HFT-All.

Table 2 shows the comparison results on mini-ImageNet. We can see that the accuracy of HFT-Last1/HFT-All is consistently higher than its corresponding accuracy of FT-Last1/FT-All under all sample sizes, layer frozen policies, and pretraining methods. Compared with FT-Last1/FT-All, HFT-Last1/HFT-All has an average performance improvement of 2.30% on the whole, which proves the effectiveness of combining the advantages of FSLDA and AFT. In addition, the results show that the average performance gains of the layer frozen policy “Last1” are higher than those of the layer frozen policy “All” (3.83% vs. 1.90%, 2.36% vs. 1.19%, and 1.38% vs. 0.86%). Since HFT-Last1 is indeed FSLDA, this phenomenon validates that the linear classifier constructed by FSLDA is much better than that acquired by fine-tuning. Thirdly, for sample size from 1-shot to 30-shot, HFT-Last1/HFT-All achieves an average performance improvement of 1.78% ∼ 2.85% over FT-Last1/FT-All, and the gains are relatively close, indicating that the proposed algorithm has good generalization ability for different sample sizes. Lastly, we can see that the accuracy of the layer frozen policy “All” is always higher or not less than its corresponding accuracy of the layer frozen policy “Last1,” which is consistent with the conclusions of [33, 34].

For tiered-ImageNet dataset, the category correlations between the training set and the test set are weak, and thus, it is more suitable for testing the generalization ability to novel few-shot classification tasks. The comparison results are shown in Table 3. Overall, we can see an average performance improvement of 2.78% for HFT-Last1/HFT-All, surpassing the average gain of 2.30% on mini-ImageNet. This shows that the proposed algorithm has strong generalization ability and can better adapt to novel few-shot classification scenarios. For layer frozen policies “Last1” and “All”, HFT-Last1/HFT-All achieves an average performance improvement of 2.66% ∼ 3.58% and 1.45% ∼ 1.77%, respectively, which is slightly larger than that on mini-ImageNet. For different sample sizes, HFT-Last1/HFT-All achieves an average performance improvement of 2.13% ∼ 3.37%. The average gains in 1-shot and 5-shot are larger than those in 10-shot, 20-shot, and 30-shot, which further illustrates that FSLDA plays an essential role when the sample size is less than 5. As for the comparison of different fine-tuning policies under the same pretraining method and the same finetuning strategy, the policy “All” is always better or not less than the policy “Last1,” which is the same as the conclusion on mini-ImageNet.

### 4.4. Ablation Experiments

In this section, we analyze the effects of FSLDA and AFT modules in our HFT, respectively. The experiments are carried out on mini-ImageNet under the two layer frozen policies “Last1” and “All,” employing the Meta-Baseline pretraining method [10]. The results are shown in Table 4. For the layer frozen policy “Last1,” HFT is indeed FSLDA; thus, AFT is useless (✓/×) when FSLDA is employed (✓). For the layer frozen policy “All,” the acquired hands-on hybrid fine-tuning strategy is built on FSLDA; thus, AFT cannot be run separately.

We can see that using FSLDA alone can perform consistently better than traditional fine-tuning methods under different sample sizes and layer frozen policies. For the layer frozen policy “Last1,” FSLDA alone achieves 2.26%, 4.35%, 4.03%, 2.82%, and 1.88% gains under the sample sizes of 1-shot, 5-shot, 10-shot, 20-shot, and 30-shot, respectively. Overall, it has an average performance improvement of 3.07%. For the layer frozen policy “All,” FSLDA also achieves gains of 3.34%, 5.47%, 4.13%, 1.54%, and 0.45% under the corresponding sample sizes though FSLDA is only designed for the last layer. Moreover, it obtains an average increase of 2.99% on the whole, which is close to that under the layer frozen policy “Last1.” A common explanation for this is that fine-tuning the classifier of the model using few-shot samples in the support set usually converges to a suboptimal solution, leading to the fine-tuned model's poor performance. FSLDA gives the classifier an optimal solution by fully excavating the professional knowledge of the novel classes, which means the FSLDA model outperforms the model with the experience-based fine-tuning method, even without fine-tuning. For the layer frozen policy “All,” AFT brings 0.40%, 0.99%, and 0.79% performance improvements over individual FSLDA under the sample sizes of 10-shot, 20-shot, and 30-shot, respectively, and the average gain reaches 0.72%. This is because the adaptive epoch obtained by AFT can predictably help the FSLDA model update parameters through backpropagation while preventing the model from underfitting and overfitting, which enables the model to achieve better performance than the FSLDA model alone. One interesting thing is that the accuracies of the policy “All” under sample sizes of 1-shot, 5-shot, and 10-shot are lower than those of the policy “Last1” for the traditional fine-tuning method, which is not consistent with the conclusions of [33, 34] and brings uncertainty to the choice of the layer frozen policy.

## 5. Conclusion

In this study, we have introduced a hybrid fine-tuning strategy (HFT) for FSC, including the FSLDA and AFT modules. FSLDA constructs the optimal linear classifier, and AFT outputs the hybrid fine-tuning strategy based on the FSLDA model. HFT solves the problem that the linear classifier is suboptimal under few-shot conditions and prevents the model from overfitting and underfitting by using the acquired hands-on hybrid finetuning strategy. By conducting extensive experiments, we find HFT achieves consistent performance improvements compared to traditional finetuning methods under different sample sizes, layer frozen policies, and few-shot classification frameworks. Intuitively, our HFT has enormous potential for FSC and even for few-shot learning. In the future, we will try to explore automatic learning methods of more hyperparameters for the fine-tuning stage.

## Figures and Tables

**Algorithm 1:**
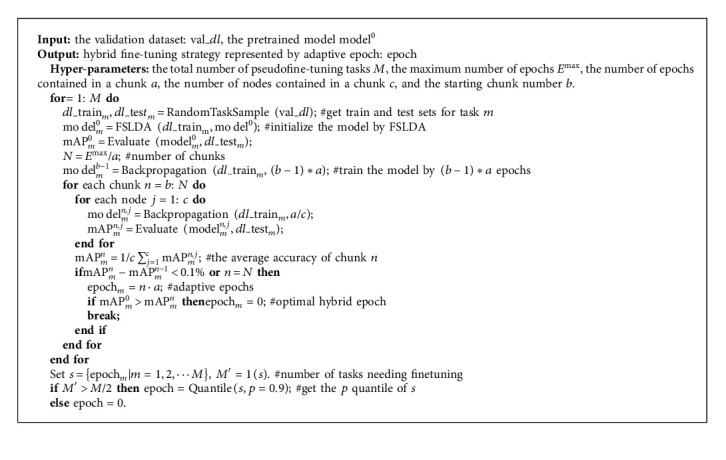
Pseudocode for the AFT module.

**Table 1:** The hands-on hybrid fine-tuning strategy acquired by the proposed method under different sample sizes and layer frozen policies.

Layer frozen policy	1-Shot	5-Shot	10-Shot	20-Shot	30-Shot
Last1	0	0	0	0	0
All	0	0	1400	1600	1800

**Table 2:** Comparison results under different pretraining methods on mini-ImageNet. “Pre-tra” and “Lay-fro” are short for the pretraining method and the layer frozen policy, respectively. We report the mean accuracy of 600 episodes and the 95% confidence intervals.

Pre-tra	Lay-fro	1-Shot	5-Shot	10-Shot	20-Shot	30-Shot	Average gain
*R2D2*	FT-Last1	50.58±0.74	66.15±0.36	71.07±0.71	75.63±0.87	76.56±0.96	**3.83**↑
	HFT-Last1	**53.47**±**0.61**	**70.13**±**0.50**	**74.72**±**0.45**	**79.67**±**0.45**	**81.16**±**0.47**	
	FT-all	51.39±0.81	68.63±0.40	73.38±0.61	79.43±0.66	80.84±0.99	**1.90**↑
	HFT-all	**53.47**±**0.61**	**70.13**±**0.50**	**75.49**±**0.59**	**81.41**±**0.70**	**82.66**±**0.38**	

*SKD-GEN0*	FT-Last1	57.83±0.53	73.91±0.53	78.19±1.03	85.03±0.76	87.01±0.45	**2.36**↑
	HFT-Last1	**60.74**±**0.68**	**77.45**±**0.49**	**81.30**±**0.38**	**86.31**±**0.42**	**87.96**±**0.39**	
	FT-all	59.94±0.77	74.34±0.56	81.69±1.09	86.96±0.32	87.43±0.74	**1.19**↑
	HFT-all	**60.74**±**0.68**	**77.45**±**0.49**	**82.34**±**1.01**	**87.15**±**0.38**	**88.63**±**0.52**	

*RFS-simple*	FT-Last1	56.99±0.60	72.43±0.32	76.27±0.29	82.97±1.29	84.02±0.94	**1.38**↑
	HFT-Last1	**58.41**±**0.71**	**73.66**±**0.51**	**78.85**±**0.45**	**83.58**±**0.56**	**85.10**±**0.55**	
	FT-all	57.10±0.21	72.79±0.59	79.31±0.28	83.01±0.51	85.23±0.91	**0.86**↑
	HFT-all	**58.41**±**0.71**	**73.66**±**0.51**	**79.69**±**0.75**	**83.81**±**0.73**	**86.16**±**0.42**	

Average gain		**2.29**↑	**2.85**↑	**2.50**↑	**1.78**↑	**2.12**↑	**2.30**↑

**Table 3:** Comparison results under different pretraining methods on tiered-ImageNet. “Pre-tra” and “Lay-fro” are short for the pretraining method and the layer frozen policy, respectively. We report the mean accuracy of 600 episodes and the 95% confidence intervals.

Pre-tra	Lay-fro	1-Shot	5-Shot	10-Shot	20-Shot	30-Shot	Average gain
*R2D2*	FT-Last1	52.10±0.70	68.99±0.70	73.21±0.30	76.82±0.89	80.38±1.24	**2.66**↑
	HFT-Last1	**55.18**±**0.72**	**72.26**±**0.66**	**75.19**±**0.62**	**80.35**±**0.63**	**81.82**±**0.62**	
	FT-all	52.90±0.78	70.87±0.69	75.02±0.28	80.04±0.72	84.69±0.96	**1.45**↑
	HFT-all	**55.18**±**0.72**	**72.26**±**0.66**	**76.57**±**0.24**	**81.50**±**0.91**	**85.24**±**0.22**	

*SKD-GEN0*	FT-Last1	60.51±0.75	76.28±0.80	80.54±0.71	83.84±0.67	86.10±0.61	**3.58**↑
	HFT-Last1	**64.17**±**0.82**	**79.42**±**0.61**	**83.75**±**0.53**	**87.60**±**0.42**	**90.25**±**0.34**	
	FT-all	61.05±0.76	76.37±0.79	83.46±0.50	87.01±1.35	90.45±1.26	**1.58**↑
	HFT-all	**64.17**±**0.82**	**79.42**±**0.61**	**83.79**±**0.89**	**87.76**±**0.98**	**91.09**±**0.93**	

*RFS-simple*	FT-Last1	60.45±0.98	74.09±0.79	78.86±0.58	83.36±0.61	83.51±1.52	**2.86**↑
	HFT-Last1	**63.76**±**0.88**	**77.74**±**0.57**	**81.27**±**0.53**	**85.35**±**0.50**	**86.45**±**0.57**	
	FT-all	60.56±0.97	75.39±0.80	80.18±0.43	83.90±0.47	88.04±1.35	**1.77**↑
	HFT-all	**63.76**±**0.88**	**77.74**±**0.57**	**81.42**±**0.81**	**84.98**±**0.65**	**89.01**±**0.81**	

Average gain		**3.37**↑	**3.37**↑	**2.41**↑	**2.51**↑	**2.13**↑	**2.78**↑

**Table 4:** Ablation experiments on mini-ImageNet employing the meta-baseline pretraining method. We report the mean accuracy of 600 episodes and the 95% confidence intervals.

	FSLDA	AFT	1-Shot	5-Shot	10-Shot	20-Shot	30-Shot
*Last1*	×	×	48.14±0.95	69.32±0.75	74.02±0.81	81.66±0.40	86.39±0.91
	✓	✓/×	**50.40**±**0.35**	**73.67**±**0.64**	**78.05**±**0.31**	**84.48**±**0.89**	**88.27**±**0.77**

*All*	×	×	47.06±0.96	68.20±0.75	73.92±0.73	82.94±0.11	87.82±0.94
	✓	×	**50.40**±**0.35**	**73.67**±**0.64**	78.05±0.31	84.48±0.89	88.27±0.77
	✓	✓	—	—	**78.45**±**0.92**	**85.47**±**1.05**	**89.06**±**0.88**

## Data Availability

The data used to support the findings of this study can be obtained from the corresponding author upon request.

## Appendix

LDA classifier: LDA is a classical optimal linear classifier using Bayes' theorem. For a *C*-way *K*-shot classification task, let *X* and *Y* be the random variables for data samples and labels, respectively. The posterior probability of an observation *x* that belongs to the *c*^th^ class can be written as

(A.1) $$\begin{array}{c} P\left(Y=c|X=x\right)=\frac{\pi_{c}f_{c}\left(x\right)}{\sum_{i=1}^{C}\pi_{c}f_{i}\left(x\right)}\text{,} \end{array}$$

where *π*_*c*_ is the prior probability which can be easily calculated by simply computing the fraction of the training observations that belong to *c*^th^ class, *f*_*c*_(*x*) is the conditional probability that an observation *x* belongs to *c*^th^ class, and ∑_*i*=1_^*C*^*π*_*c*_*f*_*i*_(*x*) is a normalization constant.

To simplify the problem, LDA assumes that *f*_*c*_(*x*) obeys multivariate Gaussian distribution and the covariance matrix Σ of all classes is the same:

(A.2) $$\begin{array}{c} f_{c}\left(x\right)=\frac{1}{2\pi^{p/2}{\left|\Sigma \right|}^{0.5}}e^{-1/2{\left(x-\mu_{c}\right)}^{T}\Sigma^{-1}\left(x-\mu_{c}\right)}\text{,} \end{array}$$

(A.3) $$\begin{array}{c} \Sigma =\frac{1}{C\left(K-1\right)}\overset{C}{\underset{c=1}{\sum }}\overset{K}{\underset{i=1}{\sum }}\left(x_{c}^{i}-\mu_{c}\right){\left(x_{c}^{i}-\mu_{c}\right)}^{T}\text{,} \end{array}$$

(A.4) $$\begin{array}{c} \mu_{c}=\frac{1}{K}\overset{K}{\underset{i=1}{\sum }}x_{c}^{i}\text{.} \end{array}$$

Thus, the posterior probability can be written as

(A.5) $$\begin{array}{c} P\left(Y=c|X=x\right)=A\cdot \pi_{c}e^{-1/2{\left(x-\mu_{c}\right)}^{T}\Sigma^{-1}\left(x-\mu_{c}\right)}\text{,} \end{array}$$

where *A*=1/∑_*i*=1_^*C*^*π*_*c*_*f*_*i*_(*x*) · 1/2^*p*/2^|Σ|^0.5^ is a constant.

Then, LDA takes the logarithm of the posterior probability (ignores the constant item):

(A.6) $$\begin{array}{c} P\left(Y=c|X=x\right)=\log\pi_{c}-\frac{1}{2}{\left(x-\mu_{c}\right)}^{T}\Sigma^{-1}\left(x-\mu_{c}\right)\text{,}\\ =\log\pi_{c}-\frac{1}{2}\left(x^{T}\Sigma^{-1}x-x^{T}\Sigma^{-1}\mu_{c}-\mu_{c}^{T}\Sigma^{-1}x+\mu_{c}^{T}\Sigma^{-1}\mu_{c}\right)\text{,}\\ =\log\pi_{c}-\frac{1}{2}\left(x^{T}\Sigma^{-1}x-2\mu_{c}^{T}\Sigma^{-1}x+\mu_{c}^{T}\Sigma^{-1}\mu_{c}\right)\text{,} \end{array}$$

where *x*^*T*^Σ^−1^*x* is independent of the category of *x*. Therefore, the linear score function can be represented as

(A.7) $$\begin{array}{c} P\left(Y=c|X=x\right)=\log\pi_{c}+\mu_{c}^{T}\Sigma^{-1}x-\frac{1}{2}\mu_{c}^{T}\Sigma^{-1}\mu_{c}\text{.} \end{array}$$

For a *C*-way *K*-shot classification task, *π*_*c*_ is also an irrelevant item and the final linear classifier function becomes

(A.8) $$\begin{array}{c} P\left(Y=c|X=x\right)=\mu_{c}^{T}\Sigma^{-1}x-\frac{1}{2}\mu_{c}^{T}\Sigma^{-1}\mu_{c}\text{.} \end{array}$$

Equations (A.3), (A.4), and (A.8) form the LDA classifier as used in Section 3.2.
